# Natural variation in antimicrobial activity and composition of the secreted metabolomes of the model moss *Ceratodon purpureus*

**DOI:** 10.1038/s41598-026-57343-2

**Published:** 2026-06-13

**Authors:** Liia R. Valeeva, Harrison R. Coker, Saida M. Ibragimova, Irina V. Kukharskaya, Lydia M. Bogomolnaya, Julie A. Howe, Margarita R. Sharipova, Eugene V. Shakirov

**Affiliations:** 1 Department of Biological Sciences, College of Science, Marshall University, Huntington, WV 25701 USA; 2 Department of Soil and Crop Sciences, Texas A&M University and Texas A&M AgriLife, College Station, TX 77843-2474 USA; 3 Laboratory of Agrobioengineering, Institute of Fundamental Medicine and Biology, Kazan Federal University, Kazan, 420021 Russia; 4 Department of Biomedical Sciences, Joan C. Edwards School of Medicine, Marshall University, Huntington, WV 25755 USA

**Keywords:** Bryophytes, Exudate, Bioactivity, Antibacterial compounds, Fire moss, Plant metabolite, Biochemistry, Biological techniques, Biotechnology, Microbiology, Plant sciences

## Abstract

Bryophytes have recently emerged as potentially promising producers of bioactive metabolites with multiple applications in biomedicine and biotechnology. Our previous studies have established that the model moss *Ceratodon purpureus* produces novel compounds with potent antimicrobial activities against Gram-positive bacteria. Here, we conducted a comparative analysis of metabolites produced by four different natural isolates of *C. purpureus* and observed considerable variation in the composition of antibacterial compounds. Next, we partially purified bioactive compounds from extracellular exudates produced by the two model *C. purpureus* lines, GG1 and R40. Untargeted metabolomic analysis revealed that GG1 isolate secreted a much more diverse set of metabolome components, including various amines, fatty acids, lactones, quinones and terpenoids, in comparison to the R40 isolate. However, the relative percentages of nitrogen-, phosphorus- and sulfur-containing compounds were notably similar in both metabolomes. Importantly, our analysis predicted the presence of several classes of potentially bioactive metabolites in metabolomes of both moss isolates. Taken together, our experiments provided an important evidence that extracellular metabolomes of the model moss *Ceratodon purpureus* are characterized by a remarkable intraspecific variability in the secretion, composition and abundance of different extracellular compounds.

**Supplementary Information:**

The online version contains supplementary material available at https://doi.org/10.1038/s41598-026-57343-2.

## Introduction

Plants produce a plethora of various bioactive compounds collectively providing a vastly underexplored niche for novel drug discovery, with potentially numerous applications in agriculture and pharmaceutical industry. Specifically, plants synthesize unique secondary metabolites with antibacterial, antifungal, antiviral, antioxidant, anti-inflammatory, antitumor and many other activities^1–3^. Up to 28% of the current arsenal of pharmaceutical tools used to treat human diseases is based on bioactive compounds from plants or their derivatives^4^. The best-known therapeutic agents of plant origin were initially isolated from flowering plants *Artemisia annua* L., *Camptotheca acuminata* Decne., *Gingko biloba* L., *Curcuma longa* L., *Podophyllum peltatum* L., *Taxus brevifolia*, *Taxus baccata*, *Combretum caffrum*, and *Euphorbia peplus*, with the majority of these pharmaceuticals typically used for anticancer and antimicrobial therapies^5^. According to the National Cancer Institute, over 35,000 plant species have been evaluated for the presence of potential antitumor compounds, with promising anticancer activity detected in over 3,000 of these species^6^. Known plant-produced classes of anticancer compounds include lignans, anthocyanins, flavonoids, coumarins, and catechins^6^. While the rising occurrence of antibiotic resistance of pathogenic microorganisms represents a major problem in the biomedical field, the secondary metabolites of plant origin hold a major promise in countering this epidemic. To tackle the problem of antibiotic resistance more broadly, over 1,000 studies since 2012 have evaluated different plant species for the production of new antibacterial agents^7^.

Plants historically used in traditional folk medicine are also being repurposed for new applications in the biopharmaceutical industry. New and more advanced methods in chromatography, mass-spectrometry and metabolomics are now making it possible to purify and identify specific active components and metabolites from relatively crude plant extracts. One of the best-known examples of metabolites purified from plants is artemisinin, a naturally occurring compound from *Artemisia annua.* While this plant has been used in traditional Chinese medicine for centuries to treat malaria infections caused by *Plasmodium falciparum*, the discovery of its active component, artemisinin, has allowed the development of the first-line treatment for malaria worldwide and has saved millions of lives since the 1990s. In addition, studies of artemisinin and its derivatives have identified their potential to treat many other human diseases, including cancer, diabetes, and viral infections^8,9^. Prostratin, a compound isolated from the bark of the mamala tree (*Homalanthus nutans*), is successfully being used for the treatment of HIV infections^10,11^. Another well-known plant-derived metabolite is Paclitaxel (Taxol), an anticancer taxane diterpene isolated from Western yew (*Taxus brevifolia* Nutt.)^4^.

Phylogenetically, the evolutionary lineage of land plants (Embryophytes) that researchers typically mine for bioactive compounds consists of up to 400,000 species from four major groups: angiosperms (flowering plants), gymnosperms (conifers, cycads, and ginkgos), seedless vascular plants (ferns, clubmosses, and horsetails) and bryophytes (non-vascular plants including mosses, liverworts, and hornworts). Of all these plant lineages, angiosperms are the most species-rich and historically have been the most commonly studied lineage to evaluate for the presence of bioactive phytochemicals^7,12^. More recently, bryophytes (the second most species-rich group among embryophytes) have been recognized for their potential to produce unique bioactive compounds^13^. These relatively small plants appear to be highly adapted to diverse and often extreme environmental conditions. This feature can be exemplified by the desert moss *Syntrichia caninervis*, which can survive in various harsh environments due to its high desiccation tolerance, adaptation to ultra-low temperatures, and resistance to anoxic conditions and gamma irradiation^14^. The renewed attention to non-vascular plants allowed scientists to evaluate genomes and transcriptomes of several bryophyte species, including the model mosses *Physcomitrium patens* and *Ceratodon purpureus*^15–17^, which are characterized by the availability of well-developed gene inactivation techniques, easy and low-cost growth and propagation methods in laboratory conditions, and dominant haploid lifecycles.

Several past studies of tissue extracts from bryophytes revealed that these plants produce many unique intracellular compounds with bioactive properties. However, the main hurdle in the field is the current lack of understanding of the molecular nature and chemical composition of these and other bioactive metabolites from bryophyte extracts. In recent years, just a handful of studies have focused on the identification of peptides from extracellular proteomes and secretomes produced by the model moss *P. patens*^18,19^. While these studies were very informative and extended our current understanding of the nature and composition of secreted moss polypeptides, flowering plants are known to exude many other classes of metabolites into the external environment, including individual amino acids, sugars, phenolics, organic acids, and other high molecular weight compounds (i.e., complex carbohydrates)^20–23^. Some of these extracellular plant metabolites are also responsible for plant-to-plant communication and for the protection against phytopathogens^24^. Moreover, in contrast to intracellular metabolites, secreted bioactive compounds are usually easier to purify and often have better stability under standard laboratory conditions. Unfortunately, our lack of understanding of the overall range and molecular composition of secreted moss metabolites stymies further progress on their potential development into future pharmaceutical products. This information would be particularly useful for model species of mosses, as the availability of their genomic and transcriptomic information can help decipher complex biosynthetic pathways utilized for the production of bioactive compounds and to enhance current efforts to utilize these plants as biofactories suitable for metabolic engineering and biotechnology.

One of the most well-studied representatives of the model bryophytes is a cosmopolitan moss *Ceratodon purpureus* (also known as the fire moss), which is able to withstand extreme temperature fluctuations. This moss shows natural resistance to dehydration and rehydration cycles, high levels of ultraviolet radiation, and can grow on poor soils contaminated with heavy metals^25^. *C. purpureus* is also adapted to diverse environmental conditions, with different *C. purpureus* populations growing in distinct natural habitats with different climate patterns from tropical forests to Antarctica^26^. Our recent study indicated that when *C. purpureus* is grown in liquid culture, it secretes extracellular compounds that inhibit the growth of Gram-positive bacteria including key human pathogens, such as *Staphylococcus aureus* and *Enterococcus faecium*^27^. However, the nature of these bioactive moss compounds remains undiscovered. Here, our goal was to determine for the first time the general composition of secreted metabolites from the *Ceratodon purpureus* moss and discover its biologically active compounds. Utilizing exudates from two novel *C. purpureus* lines B150 and B190 originating from the same geographic location (Alaska, USA), we evaluated their antibacterial activity and additionally performed metabolite analysis of the biologically active exudates from the two model *C. purpureus* lines, GG1 and R40.

## Results

### Natural variation in the production and activity of *C. purpureus* antibacterial compounds

**Fig. 1:**
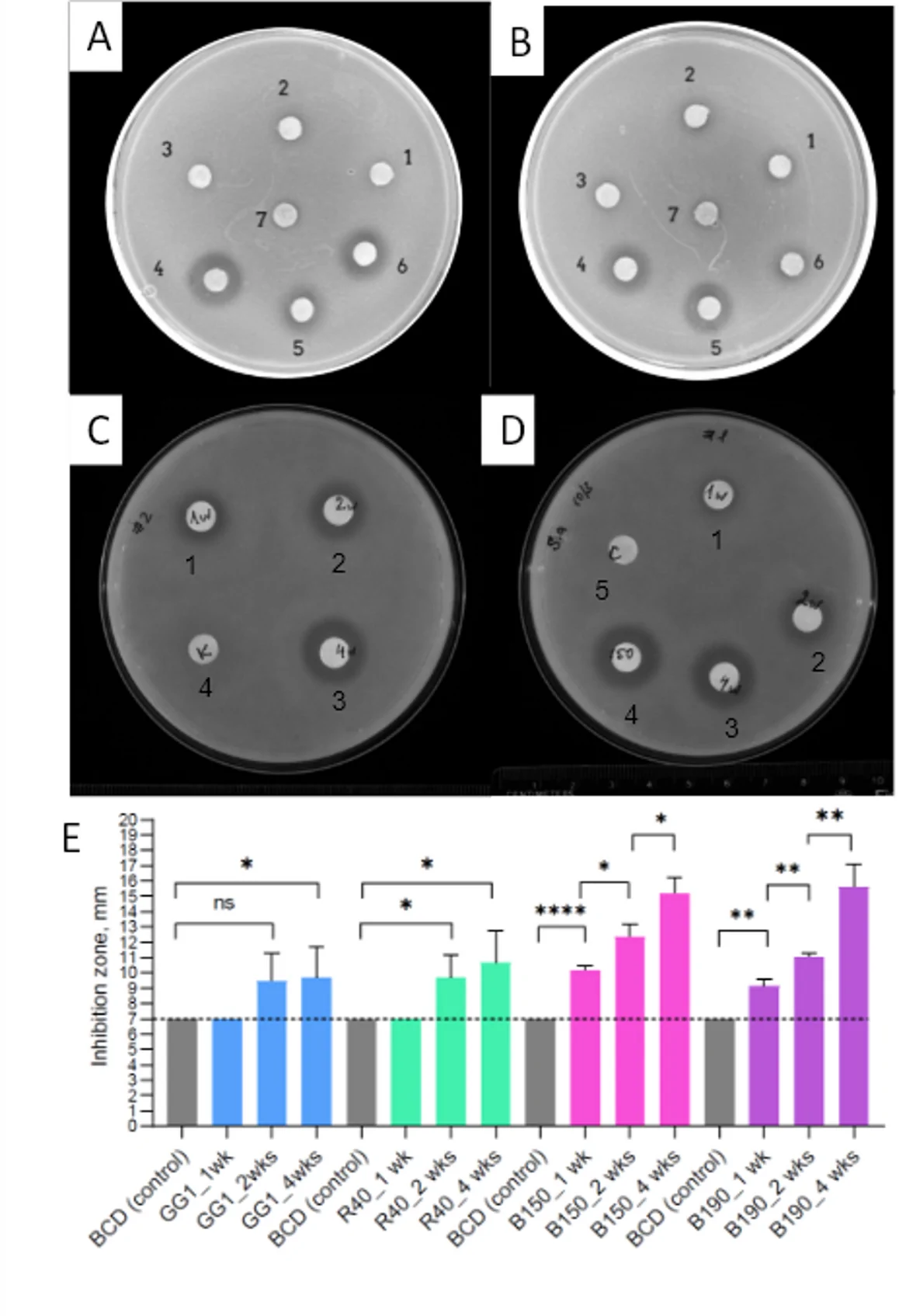
Antibacterial activity of raw exudates from *C. purpureus* isolates. Representative pictures of qualitative DDM assays with cellulose disks soaked with raw exudates (32 mg/disk) from isolates GG1 (**A**), R40 (**B**), B150 (**C**), and B190 (**D**) placed on top of *S. aureus* ATCC25923 bacterial lawns. (**A**,** B**), 1–3: disks containing secreted metabolites from 1-week-old GG1 (**A**) and R40 (**B**) isolates; 4–6: disks containing secreted GG1 and R40 metabolites after four weeks of growth; 7- negative control (BCD medium alone). (**C**), disks containing secreted metabolites from B150 isolate after 1, 2, and 4 weeks of growth (disks 1–3, respectively); 4 - negative control (BCD medium alone). (**D**), disks containing secreted metabolites from B190 isolate after 1, 2, and 4 weeks of growth (disks 1–3); 4, disk containing 4-weeks-old B150 exudate is shown for comparison; 5 - negative control (BCD medium alone). (**E**) Diameter of *S. aureus* growth inhibition area (halo) around each disk containing secreted *C. purpureus* GG1, R40, B150, and B190 metabolites after one, two, and four weeks of growth was measured and plotted. BCD (control) - negative medium control. The dotted line indicates diameter of the cellulose disk (7 mm). Data represent the means from at least 3 independent experiments and a standard deviation. The asterisks indicate significance in an unpaired t-test; statistical significance * *p* ≤ 0.05; ** ≤ 0.01; **** ≤ 0.001; ns, *p* > 0.05.

While extracellular *C. purpureus* exudates were previously shown to harbor potent antimicrobial activity against Gram-positive bacteria^27^, the extent of natural variation in this activity between different *C. purpureus* isolates is unknown. We evaluated two recently characterized moss lines originating from Alaska, USA: B150 (female) and B190 (male). When tested against *S. aureus* ATCC 25923 using disk diffusion method (DDM), exudates from both isolates grown for as little as 1 week in liquid culture displayed high antibacterial activity (Fig. 1, Supplementary Table S1). This observation is in sharp contrast with exudates from R40 and GG1 isolates, which start producing antibacterial compounds only after 2 or more weeks of growth in liquid culture (Fig. 1E, Supplementary Table S1). Furthermore, antibacterial activity in B150 and B190 exudates was consistently higher than that produced by GG1 and R40 isolates at each corresponding week, with inhibition zone reaching over 15 mm for exudates from both B150 and B190 isolates after 4 weeks of growth (Supplementary Table S1). Finally, with the levels of antibacterial activity in R40 and GG1 exudates largely stabilizing at 2–4 weeks of moss growth, B150 and B190 isolates continued to produce statistically significant increases in activity at the corresponding time points (Fig. 1E).

To test for the presence of fungal endophytes in the axenic moss tissues and exudates, we performed semi-quantitative PCR on genomic DNA extracted from *C. purpureus* isolates R40 and GG1 using universal fungal ITS primers (see Methods). No fungus-specific PCR products were detected in all analyzed samples of both moss isolates (Supplementary Figure S1). These data suggest that the antibacterial metabolites are likely to be of bryophyte origin, although the presence of endophytic fungal DNA below the PCR detection limit cannot be fully excluded. Collectively, our data emphasize the presence of substantial natural variation in the amount or stability of antibacterial activity produced by different *C. purpureus* isolates but also indicate that antibacterial activity is a universal feature of exudates from diverse *C. purpureus* strains.

### Fractionation of non-polar hydrophobic compounds with antibacterial activity from model *C. purpureus* isolates

**Fig. 2:**
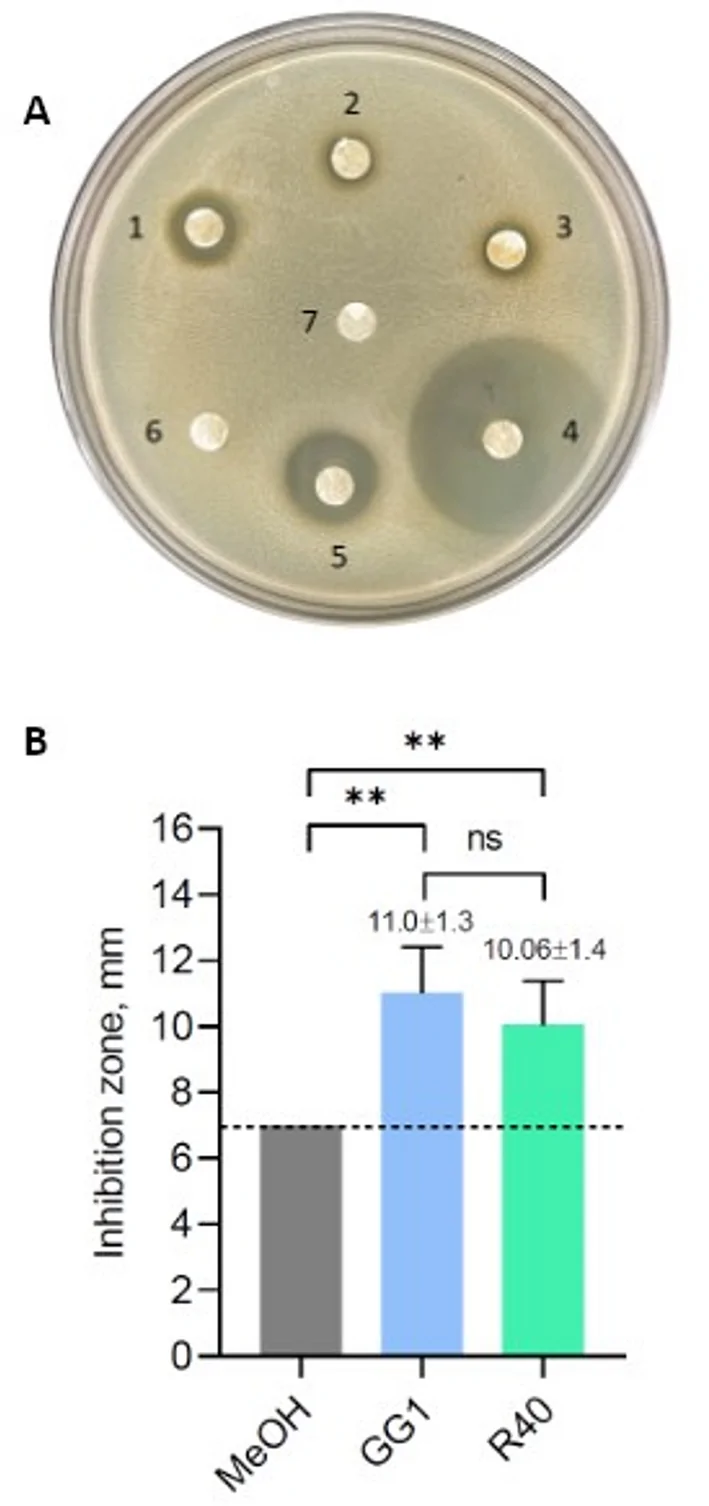
Antibacterial activity of the small-to-medium molecular weight hydrophobic fractions of extracellular compounds partially purified from 4-week-old exudates of *C. purpureus* R40 and GG1. *S. aureus ATCC* 25,923 strain was used in the analysis. **(A)** A representative image of DDM results. 1–3, disks soaked in R40 metabolites, 5 mg/disk; 4, a disk with carbenicillin (5 µg/disk, positive control); 5, a disk with chloramphenicol (5 µg/disk, positive control); 6–7, a disk soaked in 80% methanol (negative control). **(B)** Diameters of *S. aureus* growth inhibition zones (halo) around each disk containing *C. purpureus* GG1 and R40 metabolites. MeOH, 80% methanol (negative control). The dotted line indicates diameter of the cellulose disk (7 mm). Data represent the means from at least 3 independent experiments and a standard deviation. The asterisks indicate significance in an unpaired t-test; statistical significance ** ≤ 0.01; ns, *p* > 0.05.

We next focused on biochemical fractionation of antibacterial metabolites secreted by GG1 and R40 isolates, as these two model *C. purpureus* isolates feature annotated genomes and transcriptomes^17,28^, which may ultimately aid in the future bioinformatic identification of the metabolites’ biosynthetic pathways. To narrow down the pool of bioactive compounds, we utilized a nonionic sorbent Amberlite XAD16 to partially purify small-to-medium molecular weight hydrophobic molecules. DDM assays indicated that antibacterial activities against *S. aureus* were still present in Amberlite-bound fractions from both R40 and GG1 lines (Fig. 2). Notably, application of only 5 mg of the fraction enriched in hydrophobic compounds per disk from both R40 and GG1 lines led to the formation of distinct inhibition zones around disks with diameters of 10.06 ± 1.4 mm and 11.00 ± 1.3 mm, respectively (Fig. 2B), suggesting at least > 6-fold purification over activity in raw exudates (32 mg/disk).

**Fig. 3:**
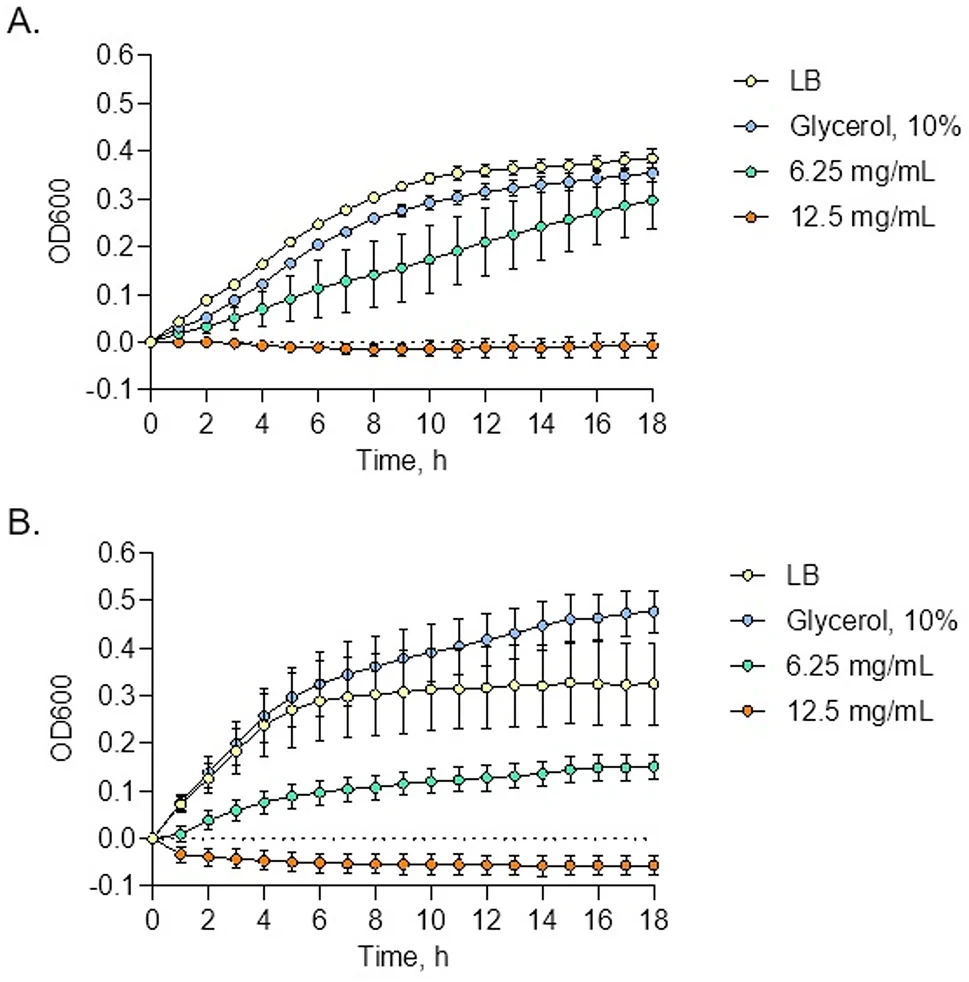
Broth microdilution method to quantitatively measure the minimum inhibitory concentration (MIC) of moss extracellular compounds. Growth of *S. aureus* ATCC 25923 in LB broth containing partially purified *C. purpureus* GG1 (**A**) and R40 (**B**) metabolites at 6.25 and 12.5 mg/mL. 10% glycerol used to dissolve dry metabolites and LB bacterium growth medium without any added compounds served as negative controls. Data represent the means from at least three independent experiments and a standard error.

To quantitatively measure the minimum inhibitory concentration (MIC) of Amberlite-bound GG1 and R40 fractions on *S. aureus* according to EUCAST protocol, we employed a broth microdilution method. For the MIC analysis, the fraction containing bioactive metabolites was dissolved in 10% glycerol instead of MeOH to avoid background bacterial growth inhibition by MeOH. Amberlite-bound fractions from both *C. purpureus* isolates fully inhibited the growth of *S. aureus* at the concentration of 12.5 mg/mL (Fig. 3A, B). The observed higher minimum inhibitory concentration of partially purified metabolites in the MIC assay in comparison to the DDM test may be largely attributed to better solubility of moss metabolites in methanol used for DDM assays in comparison to 10% glycerol. Nevertheless, our data establish that the *C. purpureus* hydrophobic exudate compounds retain their high activity against *S. aureus* cultures after Amberlite enrichment.

## Untargeted metabolite profiling of R40 and GG1 exudates reveals high intraspecific variability

**Fig. 4:**
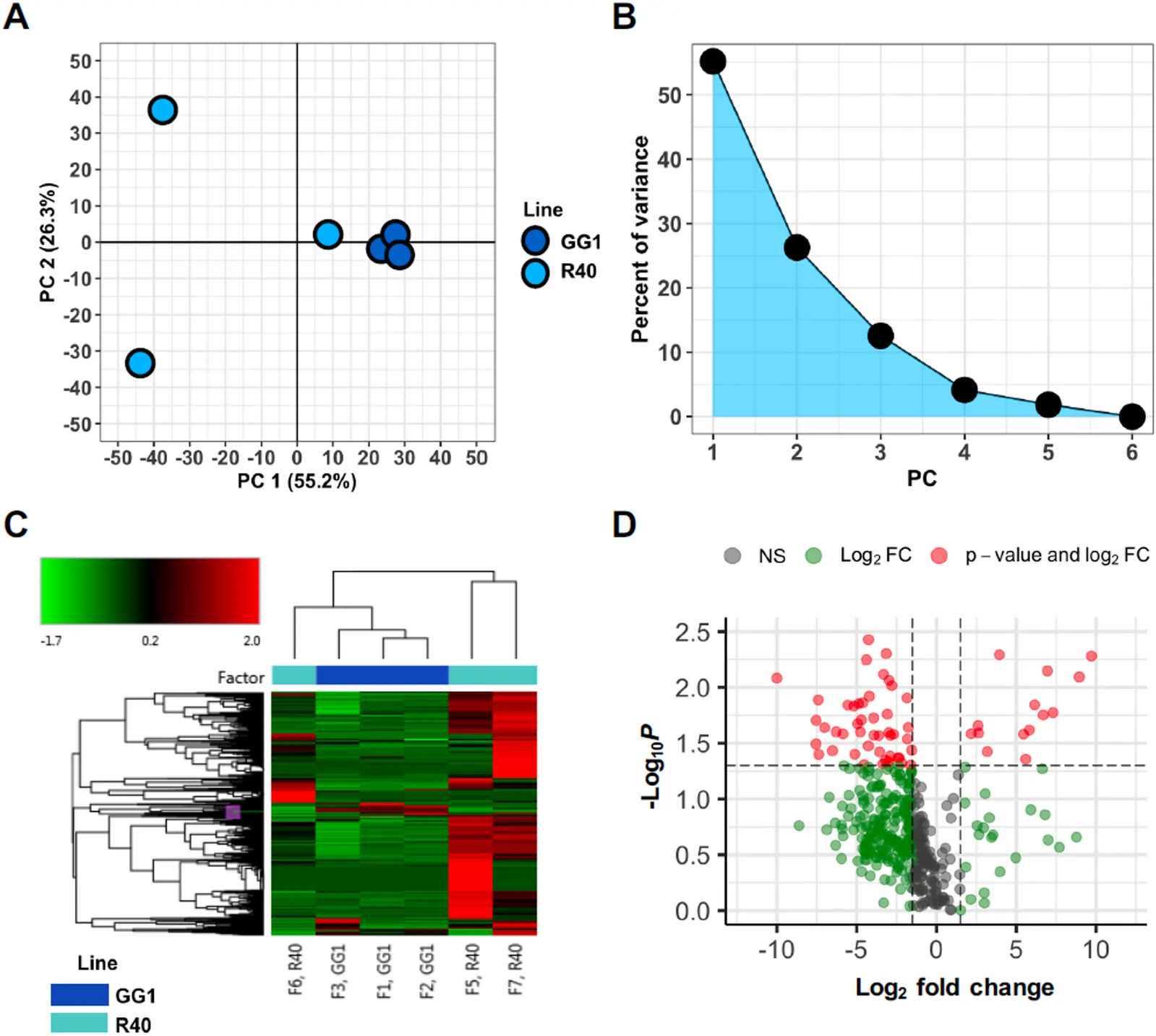
Metabolomic analysis of Amberlite-enriched *C. purpureus* GG1 and R40 exudates. (**A**) Principle component analysis of metabolite samples, with PC1 (55.2%) and PC2 (26.3%) collectively explaining 81.5% of the data variance. (**B**) Six different components explain 100% of data variance. (**C**) Heatmap evaluation of 306 metabolites identified in the analyzed libraries with dendrogram mapping of sample and metabolite similarities. Colors correspond to different values obtained after standardized treatment of different relative contents (red represents high content, green represents low content). (**D**) Volcano plot of differentially regulated (more or less abundant) metabolites with cutoffs of log_2_ FC = 1 and *P*-value = 0.05, with positive log_2_ FC indicating upregulation (higher abundance) in line R40 and negative log_2_ FC showing upregulation in GG1 line. Metabolites in the R40 and GG1 exudates were considered upregulated or downregulated (i.e., higher or lower abundance) if their FDR adjusted *P*-values were significant and if log_2_FC was > 1 or < -1, which are shown in the upper left/right sections of the volcano plot. Metabolites outside of this threshold were considered to not differ between genotypes.

We next evaluated the differences and similarities in the metabolite composition of Amberlite-bound exudate fractions from *C. purpureus* R40 and GG1 isolates by performing untargeted LC-MS/MS analysis (chromatograms reported in Supplementary Figures S2-S8). Following quality control and filtering of raw spectra, metabolomic analysis of extracellular matrices detected 1,490 features with unique m/z and individual molecular formula assignments. The global PERMANOVA model further detected a significant effect of genotype/line (*P* = 0.044), but no effect of replication indicating good experimental reproducibility (Table 1). The significant effect of genotype is explained through the PCA analysis that shows greater dispersion of R40 samples than GG1 replicates along the first and second components (explaining 81.5% of data variance) (Fig. 4A), with 6 principal components explaining 100% of the data variance (Fig. 4B). In support of the greater reproducibility of GG1 replicates, metabolites were more consistently annotated in GG1 samples than in R40 replicates, as illustrated in the heatmap (Fig. 4C).

**Table 1:** Global Permutation Multivariate Analysis of Variance (PERMANOVA) of metabolomic data. Metabolite intensity values were normalized (margin sum of squares = 1) and transformed into a Bray-Curtis dissimilarity matrix.

Factor	Df	SS	*R* ^2^	F	*P*
Genotype/Line	1	0.435	0.608	6.647	0.044
Rep	1	0.074	0.103	1.127	0.311
Line × Rep	1	0.076	0.106	1.158	0.300
Residual	2	0.131	0.183		
Total	5	0.715	1.000		

The primary difference between the two *C. purpureus* lines is a more consistent metabolite profile along with a greater number of features and compounds annotated in GG1 line compared to R40 (Table 2). GG1 contained 1,324 unique m/z and 290 annotated compounds, while R40 contained only 166 unique m/z and 43 compounds (Table 2, Supplementary Data 1). Volcano plot analysis revealed 50 compounds and 223 annotated features as upregulated (i.e., higher abundance) in GG1 and 17 compounds and 32 unannotated features upregulated in R40 (Fig. 4D; Table 2). Despite the overall greater diversity of unique chemical features found in GG1 over those in R40, the ratio of nitrogen-, phosphorus- and sulfur-containing features to total detected features was highly similar between the two lines (Table 2; Supplementary Data 1). Overall, we detected 20 different classes of compounds in GG1 metabolome versus 13 classes in the R40 line (Table 3). Further analysis indicated that in both *C. purpureus* lines, the predominant classes are heterocyclic and nitrogen-containing compounds and organic acids. Interestingly, amines, amino acids, nucleotides and fatty acids were abundant in the GG1 line but completely absent or rare in R40 (Table 3, Supplementary Data 1). Additional pathway analysis performed using the KEGG database revealed metabolic pathways with the highest number of putatively annotated compounds, including the amino acid and nucleotide metabolism, specifically, purine synthesis, followed by biosynthesis of secondary metabolites (Supplementary Table S2).

**Table 2:** Metabolomic analysis data for the GG1 and R40 lines.

Metabolome features	GG1	R40
Total number of unique m/z *	1,324	166
Unannotated features	1,034	123
Compounds	290	43
Upregulated unannotated features	223	32
Upregulated compounds	50	17
Total number of N- *	918 (69.3%)	115 (69.3%)
Total number of P- *	415 (31.3%)	57 (34.3%)
Total number of S- *	161 (12.1%)	15 (9%)

*indicates compounds and unannotated features.

**Table 3:** Classes of putatively annotated metabolites with differential abundance detected in the R40 and GG1 lines of *C. purpureus*.

Class	GG1	R40
Organic acids	52	9
Heterocyclic compounds	28	8
Amines	22	-
Amino acids	19	1
Fatty acids	12	-
Nucleotides	11	-
Amides	9	-
Phenols	6	1
Nitriles	5	-
Eicosanoids	5	5
Aldehydes	5	1
Lactones	5	-
Ketones	5	1
Furans	4	2
Alkaloids	3	1
Quinones	3	-
Terpenes	2	-
Organophosphates	1	2
Vitamins	2	1
Terpenoids	1	-
Steroid hormones	-	1
Arenes	-	1

The KEGG DRUG database was used to detect putative compounds with potential biological activities present in the metabolomes of *C. purpureus* isolates. Several promising metabolites with potentially interesting bioactivity were detected in GG1 exudates: two compounds with molecular masses similar to the synthetic sulfamethazine and sulfisomidine molecules (both have antibacterial activity)^29,30^, isopropyl myristate (emollient)^31^, and olanzapine (cardiovascular, antipsychotic and anti-allergic agent)^32^(Table 4). Additionally, two putatively bioactive compounds were upregulated in R40 metabolomes: mebeverine (neuropsychiatric agent)^33^ and vanillin (anti-inflammatory, antioxidant, and anticonvulsant agent)^34,35^(Table 4). Bioactive potential of other putatively annotated moss compounds was also predicted using the PASS (Prediction of Activity Spectra for Substances) software (Table 5; Supplementary Data 2, 3). GG1 exudates harbored 13 compounds with potential membrane disruption activity and 5 compounds with potential antineoplastic activity, while R40 exudates harbored 4 and 2 corresponding compounds, respectively. Antimicrobial (including antibacterial, fungicide, antiviral, and anti-infectious) activity was predicted for 8 and 4 annotated compounds from GG1 and R40 fractions, respectively (Table 5). Specifically, for the R40 line, compounds similar to thromboxane B2 (a possible fungicide), militarinon A (a fungicide and an antibacterial compound), and olivetolic acid (an antiseptic and anti-infective compound) were detected. In the GG1 line, 13 possible anti-infective compounds were detected, including antiviral compounds 2-[(2-chlorobenzyl)sulfanyl]-4,6-dimethylnicotinonitrile, N1-(1 H-indol-4-yl)cyclohexane-1-carboxamide, geranylacetone, antibiotic cyclo(leucylpropyl), (12Z)-9,10,11-trihydroxyoctadec-12-enoic acid and 13 S-hydroxyoctadecadienoic acid. Additionally, both the GG1 and R40 lines were found to contain such putative antimicrobial compounds as 4-ethylguaiacol (antiseptic), stigmatellin Y (antibiofilm compound)^36–38^, and phytosphingosine (antibacterial)^39^(Supplementary Data 2, 3). Collectively, these data indicate that *C. purpureus* isolates produce a number of secreted compounds with potentially beneficial bioactive properties.

**Table 4:** Kyoto Encyclopedia of Genes and Genomes (KEGG) DRUG analysis of Ceratodon extracellular hydrophobic fractions. Positive log_2_ fold change (FC) indicates upregulation (higher abundance) in line R40, negative log_2_ FC indicates upregulation in GG1 line.

KEGG ID	Metabolite	Log_2_ FC	Drug Information
D02436	Sulfamethazine-like compound*	-1.58	Antibacterial
D01526	Sulfisomidine-like compound*	-0.57	Antibacterial
D02296	Isopropyl myristate	-4.7	Emollient
D00454	Olanzapine	-3.89	Cardiovascular agent; antipsychotic agent; anti-allergic agent
D08160	Mebeverine	0.52	Neuropsychiatric agent
D00091	Vanillin	0.2	Anti-inflammatory, antioxidant, anticonvulsant agent

* According to molecular weight determination.

**Table 5:** Bioactive potential of putatively annotated compounds from the GG1 and R40 lines as predicted by the PASS software.

Bioactivity	GG1	R40
Membrane integrity agonist	13	4
Antimicrobial	8	4
Antineoplastic	5	2
Fibroblast growth factor inhibitor	1	0
Growth stimulant	1	1

## Discussion

Bryophytes are known to produce a plethora of unique bioactive compounds^13^. Their small size, relative ease of cultivation in laboratory conditions, and well-developed molecular genetics and genomics tools highlight the extraordinary potential of model bryophytes for plant biotechnology and bioengineering. Our previous studies indicated that model mosses *P. patens* and *C. purpureus* produce extracellular exudates with potent antibacterial activity against various Gram-positive bacteria^27,40^. Here, we analyzed exudates from two novel *C. purpureus* lines B150 and B190 originating from the same geographic location (Alaska, USA) and detected abundant antibacterial activity similar to the levels observed in the model GG1 and R40 *C. purpureus* lines. We observed no statistically significant differences in metabolite activity between the B150 (female) and B190 (male) lines, allowing us to conclude that the production of antibacterial compounds is a common feature of multiple *C. purpureus* moss lines. Furthermore, the observed qualitative and quantitative differences between isolates are more consistent with natural variation due to the geographical origin of the isolates (Alaska vs. other regions) rather than the isolates’ sex. We then partially purified secreted non-polar metabolites from GG1 and R40 isolates of *C. purpureus*, for which genome and transcriptome data are currently available^17^. Using the solid-phase sorbent (Amberlite), we obtained small-to-medium fractions of hydrophobic compounds from moss exudates and characterized their chemical composition. We confirmed that Amberlite-bound samples from both GG1 and R40 lines contained high levels of antibacterial activity, suggesting that biochemical fractionation of hydrophobic compounds resulted in the retention of higher levels of antimicrobial activity than was previously detected in crude exudates^27^, possibly due to the removal of inhibiting molecules or higher concentration of active compounds in the eluate.

The presence of antibacterial metabolites in *C. purpureus* isolates that are specific only against Gram-positive bacteria is intriguing but is not without precedent. Different Bryophyte species are indeed known to produce secondary metabolites with a relatively narrow spectrum of antibacterial specificity. A comparative analysis of extracts from 42 Bryophyte species indicated that 13 species produced moderate activity against Gram-positive *S. aureus* (including MRSA strains) and 4 species showed moderate activity against Gram-positive *Bacillus* sp. However, no Bryophyte species in that study produced any activity against Gram-negative *Pseudomonas aeruginosa*^41^. Similarly, hexane extracts of *Physcomitrium patens* and *Sphagnum falla*x mosses did not show antibacterial activity against Gram-negative *Pseudomonas syringae*^40^, and no activity against Gram-negative bacteria *Serratia marcescens* and *Salmonella* Typhimurium was detected in moss exudates from *P. patens* and *C. purpureus*^27,40^.

Our work has also established both substantial differences and unexpected similarities in the composition of secreted Amberlite-bound metabolome fractions of the two model *C. purpureus* isolates, GG1 and R40. On one hand, untargeted metabolomic analysis of hydrophobic fractions indicated that the GG1 isolate produced a much more diverse pool of secreted metabolites in terms of the total number and class composition of annotated metabolome components in comparison to the R40 isolate. The most distinctive feature of the GG1 metabolome was the presence of amines, fatty acids, nucleotides, amides, nitriles, lactones, quinones, terpenes and terpenoids. On the other hand, we also observed interesting similarities between metabolite classes produced by both moss isolates. Specifically, despite the much lower number of metabolites detected in the R40 secretome, the relative percentages of nitrogen-containing, phosphorus-containing and sulfur-containing compounds were remarkably similar in both metabolomes, with the largest number of annotated molecules being classified as nitrogen-containing compounds (69.3% for both lines). In general, the most represented classes of compounds for both lines were organic acids and heterocyclic compounds. Interestingly, PASS analysis program predicted the presence of several putatively bioactive compounds in both lines. Detection of these exudate components represents a substantial step forward towards better understanding of the full composition and bioactive potential of secreted moss metabolomes.

While a number of previous studies characterized intracellular metabolomes of various mosses^42–46^, including metabolites from *C. purpureus* extracts^26,47^ and compounds thought to be associated with fungal endophytes^48^, very few prior analyses focused specifically on moss secretomes. One notable exception was a series of proteomic studies that identified extracellular peptides produced by *P. patens*^49–51^, some of which were produced in response to the treatment of moss with chitosan, a derivative of chitin^19^. However, prior to our analysis, no other study characterized non-peptide extracellular compounds secreted by *C. purpureus* or other model mosses.

While the studies of moss extracellular metabolomes are still rare, many more metabolomic studies have been performed for root exudates of vascular plants. One such analysis of root exudates from 19 parental accessions used to generate *Arabidopsis thaliana* MAGIC recombinant inbred population discovered a number of similar metabolites produced by different accessions, but it also detected some substantial differences in the patterns of metabolite production associated with genetic polymorphism in the parental lines^52^. Another study of *A. thaliana* Col-0 root exudate composition detected substantial chemical diversity that included nucleosides, deoxynucleosides, aromatic amino acids, anabolites and catabolites of glucosinolates, dipeptides, indolics, salicylic and jasmonic acid catabolites, coumarins, mono-, di- and trilignols, hydroxycinnamic acid derivatives and oxylipins^53^. Thus, nitrogen-containing compounds represent a common group of metabolites often found in secretomes of flowering plants. Furthermore, many metabolites appear to belong to the “core” metabolome of plant secretomes, as demonstrated by the analysis of root exudates from three evolutionary distinct angiosperm species: *A. thaliana*, *Brachypodium distachyon*, and *Medicago truncatula*^54^.

*C. purpureus* is a cosmopolitan moss species capable of adjusting to various environmental parameters, with different isolates adapting to local growth conditions^26^. Thus, the observed differences in extracellular metabolomes between R40 and GG1 isolates may be associated with natural within-species variability in the production of secondary isolate-specific metabolites. Another possible explanation for the presence of substantial natural variation in metabolome composition may be associated with sex differences between the strains. *C. purpureus* is a dioecious species where multiple sex chromosome associated genomic and transcriptomic differences between the male R40 and the female GG1 isolates were observed^17^. Specifically, genes involved in the metabolism of amines, organophosphates, phospholipids, and inosine monophosphates were expressed at higher levels in the R40 line. On the other hand, the GG1 line is characterized by the higher expression of a group of genes necessary for secretion, regulation of macromolecule localization, transmembrane transport, amide transport, biosynthesis of the amino acids lysine and aspartic acid^17^. Thus, our discovery of a larger set of compounds secreted by the GG1 line in comparison to R40 line is consistent with the predicted overall higher rates of secretion in the GG1 line.

Interestingly, sex chromosome-dependent metabolic variations have also been detected in other dioecious plants. Using Direct Analysis in Real Time Mass Spectroscopy (DARTMS) approach, the metabolome of leaves from betel plants (*Piper betle*) was shown to substantially differ between the female and male lines^55^. Also, a comparative analysis of the metabolomes of leaf extracts from the two sea buckthorn species (*Hippophae rhamnoides* L. and *H. salicifolia* D.Don) has also discerned major variations in metabolite composition between the male and female plants^56^. Metabolomes of papaya roots from female and male plants have also showed considerable differences, with major impacts on root-associated rhizobiome composition. Specifically, nitrogen-fixing bacteria were found to be predominantly associated with the roots of the male papaya plants, while *Candidatus solibacter* and *Tumebacillus* species were mostly found in the rhizosphere of female plants^57^.

## Conclusions

Here, we performed the comparative analysis of extracellular compounds produced by natural isolates of *C. purpureus* to reveal intraspecific variation in metabolome composition and characterized their bioactive potential. Exudate fractions from all four analyzed moss isolates exhibited antibacterial activity against *S. aureus*, suggesting that the presence of secreted antibacterial compounds is a universal feature of multiple moss lines. Untargeted metabolomic analysis of bioactive Amberlite-bound secreted hydrophobic compounds from two *C. purpureus* isolates, GG1 and R40, revealed substantial differences in metabolite profile composition and abundance between the two lines. To the best of our knowledge, our study represents the first analysis of the general composition of secreted metabolites in *C. purpureus*. Taken together, our data indicate that the model moss *Ceratodon purpureus* is characterized by a remarkable intraspecific variability in the secretion, composition and abundance of different extracellular compounds. Furthermore, these data will serve as a foundation for the much-needed identification and characterization of novel bioactive antimicrobial compounds secreted by mosses and other Bryophytes. Ultimately, better understanding of moss metabolomes may lead to more efficient technologies to produce potent biopharmaceuticals for medicine and agriculture.

## Methods

### Ceratodon purpureus cultures

Axenic cultures of the green moss *C. purpureus* isolates R40 (male, NY, USA), GG1 (female, Gross Gerungs region, Austria), B150 (female, AK, USA), and B190 (male, AK, USA) were provided by Stuart McDaniel (University of Florida, USA). Moss protonemata were grown in Petri dishes on BCD agar nutrient medium (0.7% agar, pH 5.8) containing 1 mM CaCl_2_, 1.84 mM KH_2_PO_4_, 1 mM MgSO_4_, 10 mM KNO_3_, 0.045 mM FeSO_4_ and trace elements: 0.231 mM CoCl_2_ .6H_2_O, 2.2 mM CuSO_4_.5H_2_O, 9.93 mM H_3_BO_3_, 1.96 mM MnCl_2_.4H_2_O, 0.191 mM ZnSO_4_.7H_2_O, 0.169 mM KI, 1.03 mM Na_2_MoO_4_.2H_2_O, 5.5 mM ammonium tartrate^58^. Protonemata were homogenized using a T10 disperser (IKA, Staufen im Breisgau, Germany), plated on Petri dishes with BCD medium (covered with cellophane disks) and grown under standard conditions (photoperiod 16 h day/8 hours night, temperature 20–22 ºС, air humidity 45–60%) for two weeks. For metabolite analysis, protonemata were collected from plates, homogenized and transferred into 3 L of liquid BCD medium in a 5 L glass jar, and grown with stirring on a magnetic stirrer at 22 °C under a 16 h light/8 h dark photoperiod for 4 weeks. Exudate was then separated from moss cells by filtering first through a 70-µm nylon mesh and then through a 0.45-µm syringe filter. An aliquot of exudates (30 mL) was used to check for antibacterial activity as previously described^27^. Filtered exudates were then used for the solid-phase extraction procedure.

To test for the presence of fungal endophytes in the axenic moss cultures, semi-quantitative PCR was performed on DNA extracted from moss R40 and GG1 tissues and exudates using universal ITS primers specific to fungal rDNA sequences, ITS1 F (CTTGGTCATTTAGAGGAAGTAA) and ITS2 R (GCTGCGTTCTTCATCGATGC)^59^. PCR was performed using 2x Taq Master Mix (Vazyme, Union City, CA, USA) following manufacturer recommended conditions. DNA from moss cultures (three biological replicates for both R40 and GG1 lines) was extracted using the standard CTAB buffer method^60^. A serial 10-fold dilution (from 20 ng to 0.00002 ng) of fungal DNA standards from *S. cerevisiae* strain TMY18 (#E20072, Zymo Research, Irvine, CA, USA) was used as a positive control. A range of concentrations (35–309 ng/µl) of DNA extracted from moss samples was used in the PCR.

### Isolation of the active fraction of extracellular metabolites

For each moss line, partial purification was conducted from 3 L of exudates collected from 4-week-old moss cultures in three independent replicates. Extracellular compounds were separated with the non-polar resin Amberlite XAD-16 (Sigma-Aldrich, St. Louis, MO, USA), which extracted hydrophobic organic compounds with a small-to-medium molecular weight range (MW ≤ 40,000). The sorbent was pre-washed with 1,000 mL of deionized MilliQ water per 10 g of resin and activated with methanol for 30 min. Methanol was then drained, and the resin was soaked in 500 mL MilliQ water for additional 30 min. Exudates were incubated with activated Amberlite (10 g of resin per 1 L of exudate) at 4° C for 18 h while stirring. Liquid was then drained through nylon sieves (50 μm), and the sorbent was transferred to gravity columns and rinsed with three volumes of deionized water. Sorbent-absorbed compounds were incubated with 100% methanol (w/v) for 30 min and eluted into 15 mL tubes. Eluted fractions were dried on a rotary evaporator at 30 °C and stored at -20 °C in the dark.

### Antibacterial activity analysis

Antibacterial activity of moss extracellular compounds against Gram-positive bacterium *Staphylococcus aureus* ATCC 25,923 was performed using the disk diffusion assay (qualitative analysis) and the broth microdilution method to measure the minimum inhibitory concentration (quantitative analysis).

### Disk diffusion assay

Qualitative analysis of antibacterial activity of bioactive compounds was performed using the disk diffusion method (DDM) according to EUCAST protocol (The European Committee on Antimicrobial Susceptibility Testing, https://www.eucast.org/ast_of_bacteria/disk_diffusion_methodology). Bacterial cultures were grown for 16–18 h in LB medium at 37 °C. Bacterial inoculum (CFU = 1 × 10^7^ per Petri dish) was added to 5 mL of 0.7% melted LB agar, which was then overlayed on the surface of 20 mL of 2% LB agar plate. Metabolites (32 mg of crude exudates or 5 mg of partially purified fractions dissolved in 80% MeOH) were added to a sterile 7-mm diameter disk and dried at room temperature. Disks soaked in 80% methanol or liquid BCD medium were used as negative controls. Disks soaked in metabolites and control disks were placed on inoculated agar and plates were incubated at 37 °C for 18 h. The diameter of the halo zone of bacterial growth inhibition around each cellulose disk was measured in mm. All experiments were performed in at least three biological and two technical replicates.

### Minimum inhibitory concentration

Quantitative analysis of the antibacterial activity of moss metabolites was performed by determining the minimum inhibitory concentration (MIC) according to EUCAST guidelines. Bacterial cultures were grown for 16–18 h at 37 °C in LB medium and diluted to OD_600_=0.13 (corresponds to a value of 0.5 according to the McFarland standard). Each culture was then further diluted to a final concentration of approximately 1 × 10^7^ CFU/mL and transferred to a 96-well plate. Metabolites dissolved in 10% glycerol were added to the wells at concentrations of 12.5, 6.25, 3.125, 1.56 and 0.78 mg/mL. Cultures were incubated for 22 h at 35 °C in an xMark spectrophotometer (Bio-Rad) with hourly detection of optical density (OD_600_). 10% glycerol and antibiotics carbenicillin (100 µg/mL) and chloramphenicol (34 µg/mL) were used as negative and positive controls, respectively. Bacterial cultures grown in LB medium in the absence of metabolites were used as a general control for bacterial growth. MIC was defined as the lowest concentration of metabolites that inhibited bacterial growth. All experiments were performed in three biological and two technical replicates.

## Mass spectrometry (LC-MS/MS)

### Sample preparation and analysis

For LC-MS/MS processing dried samples of isolated compounds from GG1 and R40 lines were dissolved in 100% methanol (Fisher Scientific, USA) at 1 mg/mL, the tubes were placed in an ultrasonic bath for 30 s and then centrifuged for 5 min at 4,000 g to sediment insoluble fractions. The supernatant was transferred into chromatography vials and placed in a chromatograph autosampler at a temperature of 6°C. 100% methanol was used as control. Experiments were performed in three independent biological replicates on Waters Aquity UPLC HPLC system (Thermo Scientific) coupled with a Q-Exactive HF-X mass spectrometry detector (Thermo Scientific) with an electrospray ionization source. For chromatographic separation, a Waters Acquity UPLC BEH C18, 1.7 μm, 2.1 × 100 mm column with a Waters Acquity UPLC BEH C18 VanGuard 1.7 μm 2.1 × 5 mm pre-column were used. For mobile phases, water (purified in the Milli-Q Integral 3 system), formic acid and acetonitrile (Fisher Scientific) were used. Gradient elution mode was at a flow rate of 0.4 mL/min. Column temperature was kept at 60 °C. Panoramic scanning was carried out to measure positive ions in the mass range from 100 m/z to 1200 m/z, with a resolution of 70,000; emitter voltage was set at 4.5 kV, and capillary temperature was 320 °C. For tandem scanning, the resolution was set to 17,500 in the mass range from 50 m/z to the upper limit, which is determined automatically based on the mass of the precursor. Isolation of precursor ions was carried out in a window of ± 1.2 m/z. The maximum number of ions allowed for isolation in MS2 mode was set to no more than 10, while the cutoff limit for selecting a precursor for tandem analysis was set to 5 × 10^4^ units, and the normalized collision energy (NCE) was changed stepwise^10,35,60^, with averaging of the resulting fragment spectra from the corresponding precursor ion. For tandem scanning, only z = 1 + ions were considered. The maximum accumulation time for precursor ions and fragment ions was 50 ms. The AGC value for precursors and fragment ions was set to 1 × 10^6^ and 2 × 10^5^, respectively. All measured precursors were dynamically excluded from tandem MS/MS analysis for 10 s. The obtained spectra were visualized in the Xcalibur QualBrowser program (Thermo Scientific).

### Data processing

LC-MS/MS spectra were processed using Compound Discoverer (v. 3.1, Thermo Scientific). The Select Spectra node was used with open settings and a default 1.5 S/N threshold. The Detect Compounds node was used with 2 ppm mass tolerance, 10,000 minimum peak intensity, at least 5 scans per peak, peak detection S/N threshold 1.5, remove baseline true, a gap ratio threshold of 0.35, and max peak width of 0.25. Features annotated in Compound Discoverer were defined as a mass at a specific retention time and were putatively annotated with criteria from the mzCloud database including MS/MS spectral database matching. All putatively annotated features were considered compounds (level 2)^61,62^. Analyses of pathways and putatively annotated compounds with known drug activity were performed using the KEGG database from ChemSpider^63–65^. Several annotated features, such as nylon and PEG derivatives (23 features in total), were included in the analysis but considered contaminants from the Amberlite purification process. PASS (https://www.way2drug.com/PASSOnline/index.php) software was used to evaluate the general biological potential of annotated compounds with Pa > 0.7 (probability “to be active”), which estimates the probability that the detected compound may belong to a sub-class of bioactive compounds^66^. SMILES chemical notation format was used for PASS proceeding. SMILES for compounds were analyzed by PubChem Identifier Exchange Service (https://pubchem.ncbi.nlm.nih.gov/idexchange/idexchange.cgi). The LC-MS/MS dataset MSV000100001 was deposited into the MassIVE metabolomics repository (doi:https://doi.org/10.25345/C5804XZ5N).

### Statistical analysis

Metabolomic data were analyzed using the R Studio statistical software v. 4.2.0 (R Core Team, 2022). Differential expression of metabolites was assessed using a log2 fold change > 1 or < -1 and the false discovery rate (FDR) adjusted *P*-values. Partial upregulation/downregulation (i.e., higher/lower abundance of metabolites) was evaluated using only log2 fold change. Statistical significance was defined as α < 0.05. The metabolomic dataset was transformed into a matrix using Bray-Curtis dissimilarity on a normalized basis (margin sum of squares = 1). Permutational multivariate analysis of variance (PERMANOVA) was performed on the matrix using genotype, replicate, and the interaction of genotype × rep as independent variables. PERMANOVA was performed in the community ecology “*vegan”* package^67^. All figures were created using the “*ggplot2”* R package^68^. The volcano plot was generated with the BiocManager “*EnhancedVolcano*” package^69^.

## Supplementary Information

Below is the link to the electronic supplementary material.


Supplementary Material 1


## Data Availability

The data that support the findings of this study are available in the supplementary material of this article.
