# Supplementary material for: Natural variation in antimicrobial activity and composition of the secreted metabolomes of the model moss Ceratodon purpureus
Source: Sci Rep. 2026 Jun 13;16:27055. doi: 10.1038/s41598-026-57343-2 (PMC13522532; doi:10.1038/s41598-026-57343-2)
Supplement: Supplementary file 1 [file 41598_2026_57343_MOESM1_ESM.docx]

**Natural variation in antimicrobial activity and composition of the secreted metabolomes of the model moss *Ceratodon purpureus***

Liia R. Valeeva, Harrison R. Coker, Saida M. Ibragimova, Irina V. Kukharskaya, Lydia M. Bogomolnaya, Julie A. Howe, Margarita R. Sharipova, Eugene V. Shakirov

**Supplementary Materials**

**Supplementary Table S1.** Antibacterial activity of extracellular exudates from *C. purpureus* GG1, R40, B150, and B190 lines, as measured by DDM assay.

| **Cultivation time** | **No exudate control ^a^** | **Bacterial Growth Inhibition Zone, mm** | | | |
| --- | --- | --- | --- | --- | --- |
|  |  | **GG1** | **R40** | **B150** | **B190** |
| 1 week | 7 | 7 | 7 | 10.2±0.3**** | 9.1±0.4** |
| 2 weeks | 7 | 9.5±1.8^ns^ | 9.7±1.5* | 12.4±0.8* | 11.1±0.2** |
| 4 weeks | 7 | 9.7±2.1* | 10.7±2.1* | 15.2±1.0* | 15.6±1.5** |

^a^ Cellulose disk diameter is 7 mm (no antibacterial activity). * p ≤ 0.05; ** p ≤ 0.01; **** p ≤ 0.001; ns, p > 0.05.

**Supplementary Table S2.** Kyoto Encyclopedia of Genes and Genes (KEGG) pathway analysis.

Metabolites were compared to the *Physcomitrium patens* KEGG database and the number of metabolites counted for the corresponding pathways. Positive log_2_ fold change (FC) indicates upregulation (higher abundance) in line R40, negative log_2_ FC indicates upregulation in GG1 line.

| Count | KEGG Pathway | KEGG ID | Metabolite | Log_2_ fold change |
| --- | --- | --- | --- | --- |
| 17 | Metabolic pathways | [C00020](https://www.genome.jp/entry/C00020) | AMP | -5.91 |
|  |  | [C00079](https://www.genome.jp/entry/C00079) | L-Phenylalanine | -2.99 |
|  |  | [C00082](https://www.genome.jp/entry/C00082) | L-Tyrosine | -0.85 |
|  |  | [C00147](https://www.genome.jp/entry/C00147) | Adenine | -1.44 |
|  |  | [C00212](https://www.genome.jp/entry/C00212) | Adenosine | -2.38 |
|  |  | [C00242](https://www.genome.jp/entry/C00242) | Guanine | -1.67 |
|  |  | [C00314](https://www.genome.jp/entry/C00314) | Pyridoxine | -0.8 |
|  |  | [C00319](https://www.genome.jp/entry/C00319) | Sphingosine | -1.71 |
|  |  | [C00575](https://www.genome.jp/entry/C00575) | 3',5'-Cyclic AMP | -1.67 |
|  |  | [C01152](https://www.genome.jp/entry/C01152) | N(pi)-Methyl-L-histidine | -1.58 |
|  |  | [C01226](https://www.genome.jp/entry/C01226) | (9S,13S,15Z)-12-Oxophyto-10,15-dienoate | -3 |
|  |  | [C01367](https://www.genome.jp/entry/C01367) | 3'-AMP | -5.91 |
|  |  | [C01606](https://www.genome.jp/entry/C01606) | Phthalate | -0.77 |
|  |  | [C01717](https://www.genome.jp/entry/C01717) | 4-Hydroxy-2-quinolinecarboxylic acid | -2.48 |
|  |  | [C02938](https://www.genome.jp/entry/C02938) | 3-Indoleacetonitrile |  |
|  |  | [C05961](https://www.genome.jp/entry/C05961) | 6-Keto-prostaglandin F1alpha | 7.3 |
|  |  | [C12312](https://www.genome.jp/entry/C12312) | Indolin-2-one | -1.03 |
| 7 | Biosynthesis of secondary metabolites | [C00020](https://www.genome.jp/entry/C00020) | AMP | -5.91 |
|  |  | [C00079](https://www.genome.jp/entry/C00079) | L-Phenylalanine | -2.99 |
|  |  | [C00082](https://www.genome.jp/entry/C00082) | L-Tyrosine | -0.85 |
|  |  | [C00147](https://www.genome.jp/entry/C00147) | Adenine | -1.44 |
|  |  | [C01226](https://www.genome.jp/entry/C01226) | (9S,13S,15Z)-12-Oxophyto-10,15-dienoate | -3 |
|  |  | [C09315](https://www.genome.jp/entry/C09315) | Umbelliferone | -0.94 |
|  |  | [C12312](https://www.genome.jp/entry/C12312) | Indolin-2-one | -1.03 |
| 6 | Purine metabolism | [C00020](https://www.genome.jp/entry/C00020) | AMP | -5.91 |
|  |  | [C00147](https://www.genome.jp/entry/C00147) | Adenine | -1.44 |
|  |  | [C00212](https://www.genome.jp/entry/C00212) | Adenosine | -2.38 |
|  |  | [C00242](https://www.genome.jp/entry/C00242) | Guanine | -1.67 |
|  |  | [C00575](https://www.genome.jp/entry/C00575) | 3',5'-Cyclic AMP | -1.67 |
|  |  | [C01367](https://www.genome.jp/entry/C01367) | 3'-AMP | -5.91 |
| 4 | Biosynthesis of various plant secondary metabolites | [C00079](https://www.genome.jp/entry/C00079) | L-Phenylalanine | -2.99 |
|  |  | [C00082](https://www.genome.jp/entry/C00082) | L-Tyrosine | -0.85 |
|  |  | [C09315](https://www.genome.jp/entry/C09315) | Umbelliferone | -0.94 |
|  |  | [C12312](https://www.genome.jp/entry/C12312) | Indolin-2-one | -1.03 |
| 4 | Nucleotide metabolism | [C00020](https://www.genome.jp/entry/C00020) | AMP | -5.91 |
|  |  | [C00147](https://www.genome.jp/entry/C00147) | Adenine | -1.44 |
|  |  | [C00212](https://www.genome.jp/entry/C00212) | Adenosine | -2.38 |
|  |  | [C00242](https://www.genome.jp/entry/C00242) | Guanine | -1.67 |
| 3 | ABD transporters | [C00079](https://www.genome.jp/entry/C00079) | L-Phenylalanine | -2.99 |
|  |  | [C00212](https://www.genome.jp/entry/C00212) | Adenosine | -2.38 |
|  |  | [C01606](https://www.genome.jp/entry/C01606) | Phthalate | -0.77 |
| 3 | Biosynthesis of cofactors | [C00020](https://www.genome.jp/entry/C00020) | AMP | -5.91 |
|  |  | [C00082](https://www.genome.jp/entry/C00082) | L-Tyrosine | -0.85 |
|  |  | [C00314](https://www.genome.jp/entry/C00314) | Pyridoxine | -0.8 |
| 3 | Tryptophan metabolism | [C00955](https://www.genome.jp/entry/C00955) | Indole-3-ethanol |  |
|  |  | [C01717](https://www.genome.jp/entry/C01717) | 4-Hydroxy-2-quinolinecarboxylic acid | -2.48 |
|  |  | [C02938](https://www.genome.jp/entry/C02938) | 3-Indoleacetonitrile |  |
| 2 | Alpha-Linolenic acid metabolism | [C01226](https://www.genome.jp/entry/C01226) | (9S,13S,15Z)-12-Oxophyto-10,15-dienoate | -3 |
|  |  | [C16316](https://www.genome.jp/entry/C16316) | 13(S)-HOT | -1.27 |
| 2 | 2-Oxocarboxylic acid metabolism | [C00079](https://www.genome.jp/entry/C00079) | L-Phenylalanine | -2.99 |
|  |  | [C00082](https://www.genome.jp/entry/C00082) | L-Tyrosine | -0.85 |
| 2 | Cyanoamino acid metabolism | [C00079](https://www.genome.jp/entry/C00079) | L-Phenylalanine | -2.99 |
|  |  | [C00082](https://www.genome.jp/entry/C00082) | L-Tyrosine | -0.85 |
| 2 | Aminoacyl-tRNA biosynthesis | [C00079](https://www.genome.jp/entry/C00079) | L-Phenylalanine | -2.99 |
|  |  | [C00082](https://www.genome.jp/entry/C00082) | L-Tyrosine | -0.85 |
| 2 | Zeatin biosynthesis | [C00020](https://www.genome.jp/entry/C00020) | AMP | -5.91 |
|  |  | [C00147](https://www.genome.jp/entry/C00147) | Adenine | -1.44 |
| 2 | Phenylpropanoid biosynthesis | [C00079](https://www.genome.jp/entry/C00079) | L-Phenylalanine | -2.99 |
|  |  | [C00082](https://www.genome.jp/entry/C00082) | L-Tyrosine | -0.85 |
| 2 | Phenylalanine metabolism | [C00079](https://www.genome.jp/entry/C00079) | L-Phenylalanine | -2.99 |
|  |  | [C00082](https://www.genome.jp/entry/C00082) | L-Tyrosine | -0.85 |
| 2 | Arachidonic acid metabolism | [C05961](https://www.genome.jp/entry/C05961) | 6-Keto-prostaglandin F1alpha | 7.3 |
|  |  | [C14794](https://www.genome.jp/entry/C14794) | 2,3-Dinor-8-iso prostaglandin |  |
| 2 | Biosynthesis of amino acids | [C00079](https://www.genome.jp/entry/C00079) | L-Phenylalanine | -2.99 |
|  |  | [C00082](https://www.genome.jp/entry/C00082) | L-Tyrosine | -0.85 |
| 2 | Linoleic acid metabolism | [C14766](https://www.genome.jp/entry/C14766) | 9-OxoODE | -1.27 |
|  |  | [C14829](https://www.genome.jp/entry/C14829) | 12,13-DHOME | -1.05 |
| 2 | Glucosinolate biosynthesis | [C00079](https://www.genome.jp/entry/C00079) | L-Phenylalanine | -2.99 |
|  |  | [C00082](https://www.genome.jp/entry/C00082) | L-Tyrosine | -0.85 |
| 1 | Vitamin B6 metabolism | [C00314](https://www.genome.jp/entry/C00314) | Pyridoxine | -0.8 |
| 1 | D-amino acid metabolism | [C00079](https://www.genome.jp/entry/C00079) | L-Phenylalanine | -2.99 |
| 1 | Betalain biosynthesis | [C00082](https://www.genome.jp/entry/C00082) | L-Tyrosine | -0.85 |
| 1 | Sphingolipid metabolism | [C00319](https://www.genome.jp/entry/C00319) | Sphingosine | -1.71 |
| 1 | Biosynthesis of unsaturated fatty acids | [C06429](https://www.genome.jp/entry/C06429) | Docosahexaenoic acid | -1.19 |
| 1 | Thiamin meabolism | [C00082](https://www.genome.jp/entry/C00082) | L-Tyrosine | -0.85 |
| 1 | Ubiqinone and other terpenoid-quinone biosynthesis | [C00082](https://www.genome.jp/entry/C00082) | L-Tyrosine | -0.85 |
| 1 | Monobactam biosynthesis | [C00082](https://www.genome.jp/entry/C00082) | L-Tyrosine | -0.85 |
| 1 | Tyrosine metabolism | [C00082](https://www.genome.jp/entry/C00082) | L-Tyrosine | -0.85 |
| 1 | Isoquinoline alkaloid biosynthesis | [C00082](https://www.genome.jp/entry/C00082) | L-Tyrosine | -0.85 |
| 1 | Histidine metabolism | [C01152](https://www.genome.jp/entry/C01152) | N(pi)-Methyl-L-histidine | -1.58 |
| 1 | Tropane, piperdine and pyridine alkaloid biosynthesis | [C00079](https://www.genome.jp/entry/C00079) | L-Phenylalanine | -2.99 |

**Supplementary Figure S1.** **Examination of *C. purpureus* R40 and GG1 tissues and liquid cultures for the presence of fungal DNA.** All PCR reactions were performed using universal fungal ITS primers. **(A)** Negative PCR controls: Cntr, No DNA control for reactions with moss samples; NTC, no DNA control for reactions with fungal DNA standards. For a positive PCR control, seven independent reactions were performed with commercial fungal DNA standards (lanes labeled 1 through 7 with 10-fold titration of fungal DNA from 20 ng per reaction (lane 1) to 0.00002 ng per reaction (lane 7). PCR reactions with the same fungal ITS primers were also performed with DNA extracted from 3 biological replicates each of R40 (R40_1, R40_2 and R40_3) and GG1 (GG1_1, GG1_2 and GG1_3) tissues grown on solid BCD medium. **(B)**, Agarose gel stained with EtBr with genomic DNA extracted from R40 and GG1 *C. purpureus* strains (three biological replicates each) is shown as a control. Wells contain RNase A untreated (left) and treated (right) genomic DNA from *C. purpureus* tissues. **(C)**, PCR test with the same ITS primers as in (A) was also performed on exudates of R40 and GG1 strains grown in liquid BCD medium for 4 weeks. Uninoculated BCD medium reactions (BCD1, BCD2 and BCD3) were used as negative controls. Three biological replicates of exudates from R40 (R40_Exud1, R40_Exud2, and R40_Exud3) and GG1 (GG1_Exud1, GG1_Exud2, and GG1_Exud3) strains were tested for the presence of fungal DNA.

**Supplementary Figure S2**. LC-MS/MS analysis of the R40-1 sample, total ion chromatogram (TIC)

**Supplementary Figure S3**. LC-MS/MS analysis of the R40-2 sample, total ion chromatogram (TIC)

**Supplementary Figure S4**. LC-MS/MS analysis of the R40-3 sample, total ion chromatogram (TIC)

**Supplementary Figure S5**. LC-MS/MS analysis of the GG1-1 sample, total ion chromatogram (TIC)

**Supplementary Figure S6**. LC-MS/MS analysis of the GG1-2 sample, total ion chromatogram (TIC)

**Supplementary Figure S7**. LC-MS/MS analysis of the GG1-3 sample, total ion chromatogram (TIC)

**Supplementary Figure S8**. LC-MS/MS analysis of methanol control, total ion chromatogram (TIC).
